# Detection of HBsAg mutants in the blood donor population of Pakistan

**DOI:** 10.1371/journal.pone.0188066

**Published:** 2017-11-22

**Authors:** Ahmad Farooq, Usman Waheed, Hasan Abbas Zaheer, Fahad Aldakheel, Shatha Alduraywish, Muhammad Arshad

**Affiliations:** 1 Department of Bioinformatics & Biotechnology, International Islamic University, Islamabad, Pakistan; 2 Departments of Pathology and Blood Bank, Shaheed Zulfiqar Ali Bhutto Medical University, Islamabad, Pakistan; 3 Safe Blood Transfusion Programme, Ministry of National Health Services, Government of Pakistan, Islamabad, Pakistan; 4 Department of Clinical Laboratory Medicine, College of Applied Medical Sciences, King Saud University, Riyadh, Saudi Arabia; 5 Department of Family & Community Medicine, College of Medicine, King Saud University, Riyadh, Saudi Arabia; Harvard Medical School, UNITED STATES

## Abstract

**Background:**

Infection with the Hepatitis B virus (HBV) continues to be one of the leading healthcare issues in Pakistan, affecting over 6 million people. The existence of HBsAg mutants is well documented in many countries. In Pakistan, HBV screening in the majority of the blood banks is performed by Rapid Detection Devices or ELISA tests. These tests are designed to detect HBsAg, but may not detect the mutant HBsAg. Failure to detect the HBsAg mutant may result in the transmission of HBV infection from donor to recipient. Hence, there is a need to identify a HBsAg assay which can detect mutants in a country where simple and conventional HBsAg assays with varying sensitivity and specificity are used to detect HBV infections.

**Material and methods:**

Three routinely used diagnostic tests (Rapid Detection Devices, ELISA and CLIA) for HBsAg were compared with the LIAISON^®^ XL Murex HBsAg Quant Assay to determine the prevalence of HBV mutants in the Pakistani blood donor population.

The samples of blood donors from different cities of Pakistan were collected. The testing was performed using SD Bioline rapid assay (n = 1500), ELISA (n = 1500), and Abbott ARCHITECT^®^CLIA system (n = 1500) at the centers where the donations were collected. All samples (n = 4500) were re-tested for comparative analysis on the LIAISON^®^ XL Murex HBsAg Quant assay (DiaSorin S.p.A.). PCR testing was performed as a gold standard on all discordant samples.

**Results:**

119/4500 (2.64%) of the samples were positive for antibodies against HBsAg. The sensitivity of SD Bioline Rapid, GB HBsAg ELISA, Abbott ARCHITECT^®^ and LIAISON^®^ XL Murex HBsAg Quant assay was 17.24%, 43.75%, 90.91%and 100% respectively. The specificity of SD Bioline Rapid, GB HBsAg ELISA, Abbott ARCHITECT^®^ and LIAISON^®^ XL Murex HBsAg Quant Assay was 98.82%, 99.59%, 100% and 100%, respectively.

**Conclusion:**

LIAISON^®^ XL Murex HBsAg Quant assay is a highly sensitive, specific and accurate screening assay for detecting wild type as well as mutant HBsAg.

## Introduction

Hepatitis B virus (HBV) infections are one of the leading global health issues. HBV is the causative agent resulting in severe liver infection and if particularly common in under developed countries with poor medical facilities. An estimated 240 million people worldwide are chronically infected with HBV (defined as the presence of hepatitis B surface antigen for at least 6 months) [1]. HBV has the capacity to cause both acute and chronic liver diseases after virus infection. Liver diseases, including cirrhosis and cancer claim more than 650,000 lives annually, the majority in low and middle-income countries where hepatitis B infection is leading to escalating morbidity and mortality and becoming an increasing major public health problem [2].

The transmission of HBV occurs mainly through direct contact with blood, intravenous injections, blood transfusion and sexual relations [3]. It is among main causes of transfusion associated chronic diseases, liver cirrhosis, hepatocellular carcinoma and death. Pakistan is facing an alarming increase in the incidence of hepatitis B infection with contaminated blood transfusion reported to be a major contributing factor [4].

The replication of HBV DNA is carried out with the help of RNA reverse transcriptase resulting in the synthesis of HBV variants. As the reverse transcriptase of HBV is unable to perform proof reading of newly synthesized strands, addition of mismatched base pairs may occur at higher rate. This inefficiency of proof reading leads to the generation of multiple variant transcripts, even from a single template of DNA [5].

It is estimated that the HBV displays significant genetic uncertainty with an approximate rate of 1.4–3.2 × 10^−5^ nucleotide substitutions/site each year resulting in well recognized sub-types of the virus [6]. In Pakistan, for routine diagnosis and blood donation screening, HBV infection is detected through testing for HBsAg. Majority of blood banks screen for HBV using ICT (immunochromatographic tests) or ELISA (enzyme-linked immunosorbent assay), with less than one percent of centers using DNA based detection [5, 7].

The tests currently used in routine diagnosis may result in poor sensitivity due to mutations within and/or outside of the determinants that affect conformational epitope recognition or HBsAg secretion or expression. The presence of HBsAg mutants is well documented in many countries. This may be associated with selection pressure being imposed onto the virus due to vaccination or treatment regimes.

Various HBsAg diagnostic assays target specific epitopes present in the “a” determinant (directed against the second loop of this determinant: aa 139–147) [8,9]. As the variations occur in conformational epitopes of these mutants, all diagnostic assays designed on basis of such conformational epitopes fail to detect viral infection [10, 11, 12]. Unsuccessful detection of these mutants in blood donors may result in the transfusion of HBV from donor to recipient.

In Pakistan, the prevalence of HBsAg mutants has not been studied. There is a need of time for an HBsAg assay which can detect mutants in a country like Pakistan, where simple, unreliable and conventional HBsAg assays are used to detect HBV infection. Pakistan is country of about 200 million people with an already over burdened health budget. Current HBV detection methods are already greatly compromised due to poor quality. This is an aspect of our health system that is in urgent need of reform.

## Material and methods

This prospective study was performed in the Department of Blood Transfusion Services, Shaheed Zulfiqar Ali Bhutto Medical University, Islamabad, Pakistan, a premier tertiary care hospital of the Federal Capital. Healthy blood donor samples (n = 4500) were collected in serum separator tubes from 3 major blood donation centers from 3 different cities of Pakistan including capital city Islamabad, provincial head quarter Lahore and Mirpur Azad Jammu and Kashmir, Pakistan. The blood donation center of Shaheed Zulfiqar Ali Bhutto, University Islamabad receives donators belonging to all regions of Pakistan. Serum was separated from blood by centrifugation at 5,000 rpm for 10 min at 4°C and sent to the testing laboratory. The study was approved by the ethical committee of the Department of Bioinformatics & Biotechnology, International Islamic University, Islamabad, Pakistan. The participants were verbally informed regarding the purpose of sample collection and study design. All blood donors in the study were healthy males. No female donor was studied because the ratio of female blood donors is almost nil in Pakistan.

### Inclusion and exclusion criteria

To be included in this study, blood donors needed to be aged between 18–60 years, weigh more than 50 kg each and have a haemoglobin concentration above 12 g/dl. A thorough medical history was taken to ensure donors met the inclusion criteria. The exclusion criteria used were: history of jaundice, malaria, drug addiction, anaemia, repeated transfusions and any evidence of cardiac, renal, or pulmonary disease.

### Assays

HBsAg in blood samples was measured using one of three routinely used diagnostic assays; The SD BIOLINE HBsAg WB, ELISA and Abbott ARCHITECT^®^. A total of 4500 blood samples (n = 4500) were tested for comparative analysis, of which 1500, were tested on SD Bioline rapid assay. The SD Bioline HBsAg WB is a rapid, qualitative test for the detection of HBsAg in human serum, plasma or whole blood, and is the most widely used HBV screening method in blood banks and laboratories in Pakistan. The SD Bioline HBsAg WB kit is intended only for an initial screening and is tested on reactive samples according to the manufacturer’s instructions [13]. ELISA testing was performed (n = 1500) using GB HBsAg SURASE B-96 (TMB) ELISA Kit according to the standard protocol. In human serum or plasma, the direct sandwich ELISA was used for the qualitative detection of HBsAg. Abbott ARCHITECT^®^ (Abbott Laboratories, Abbott Park, IL, USA) testing was performed using chemiluminescence immunoassays (CLIA) according to the manufacturer’s instructions.

To determine the efficacy and sensitivity of each method/system, all samples (n = 4500) were re-tested for comparative analysis on the fully automated LIAISON^®^ XL Murex HBsAg Quant assay (DiaSorin S.p.A., Saluggia, Italy) a chemiluminescence immunoassay (CLIA), according to a standard protocol. PCR testing was then performed as a gold standard on all discordant samples. DNA from serum samples was extracted using ExiPrep^™^ Dx Viral DNA Kit and viral DNA was extracted from clinical samples by using a lysis buffer to disrupt viral structure, following standard manufacturers’ protocol. The exposed genomic DNA bound to the surface of silica magnetic beads in binding buffer. AccuPower^®^ HBV Quantitative PCR Kit was used to measure the HBV-DNA in plasma. AccuPower^®^ HBV Quantitative PCR Kit is an *in vitro* diagnostic kit designed for the quantification of HBV DNA in human EDTA-plasma samples through real-time PCR using ExiStation^™^ MDx System.

### Statistical methods

Sensitivity and specificity with 95% confidence intervals (CI) of each HBsAg assay was determined. Positive predictive values (PPV) and negative predictive values (NPV) (with 95% CI) were also calculated. All the data was stored and analyzed on computer using SPSS version 11 and was presented in the form of tables.

## Results

A total of 4500 blood samples were tested for comparative analysis, of which n = 1500 were tested using the SD Bioline rapid kit, n = 1500 on ELISA and n = 1500 on Abbott ARCHITECT^®^. All (n = 1500) samples were tested on the LIAISON^®^ XL CLIA Murex assay. Of 4500 samples, 119 (2.64%) were found positive for the HBsAg. The sensitivity of SD Bioline, GB HBsAg ELISA Kit, Abbott ARCHITECT^®^ and LIAISON^®^ XL CLIA Murex assay in the population tested were found to be 17.24%, 43.75%, 90.91% and 100% respectively (Tables 1–3).

**Table 1:** Overall results of SD Bioline rapid kit (n = 1500).

Kit Method SD Bioline Rapid	Total	PCR	
		Negative	Negative
**Reactive**	27	10	17
**Non-Reactive**	1473	48	1425
**Total**	1500	58	1432
		**Value**	**95% CI**
**Sensitivity**		17.24%	8.59% to 29.43%
**Specificity**		98.82%	98.12% to 99.31%
**Positive Predictive Value**		37.04%	21.99% to 55.11%
**Negative Predictive Value**		96.74%	96.35% to 97.09%
**Positive Likelihood ratio**		14.62	7.01 to 30.52
**Negative Likelihood ratio**		0.84	0.74 to 0.94
**Prevalence**		3.87%	2.95% to 4.97%
**Accuracy**		**95.66%**	

**Table 2:** Overall results of ELISA (n = 1500).

ELISA Method	Total	PCR	
		Positive	Negative
**Reactive**	20	14	06
**Non-Reactive**	1480	18	1462
**Total**	1500	32	1468
		**Value**	**95% CI**
**Sensitivity**		43.75%	26.36% to 62.34%
**Specificity**		99.59%	99.11% to 99.85%
**Positive Predictive Value**		70.00%	48.93% to 85.03%
**Negative Predictive Value**		98.78%	98.36% to 99.10%
**Positive Likelihood ratio**		107.04	43.96 to 260.65
**Negative Likelihood ratio**		0.56	0.42 to 0.77
**Prevalence**		2.13%	1.46% to 3.00%
**Accuracy**		98.4%	

**Table 3:** Overall results of Abbott Architect^®^ (n = 1500).

Abbot Method	Total	PCR	
		Positive	Negative
**Reactive**	30	30	00
**Non-Reactive**	1470	03	1467
**Total**	1500	33	1467
		**Value**	**95% CI**
**Sensitivity**		90.91%	75.67% to 98.08%
**Specificity**		100.00%	99.75% to 100.00%
**Positive Predictive Value**		100.00%	88.64% to 100%
**Negative Predictive Value**		99.80%	99.40% to 99.93%
**Positive Likelihood ratio**		infinity	
**Negative Likelihood ratio**		0.09	0.03 to 0.27
**Prevalence**		2.20%	1.52% to 3.08%
**Accuracy**		99.8%	

The specificity of SD Bioline, ELISA, Abbott ARCHITECT^®^ and LIAISON^®^XL CLIA Murex Assay was 98.82%, 99.59%, 100% and 100% respectively (Tables 1–3).

Both quantitative and qualitative tests were performed for HBsAg detection. The quantitative performance of the above mentioned assays were compared in 4500 sera of healthy blood donors. We have found that 1.80% and 3.86% of the samples were positive for HBsAg by SD Bioline and LIAISON^®^ XL CLIA Murex Assay respectively (n = 1500). 1.30% and 2.13% were positive for HBsAg by ELISA and LIAISON^®^ XL CLIA Murex Assay, respectively (n = 1500); 2.0% and 2.30% were positive for HBsAg by Abbott ARCHITECT^®^ system and LIAISON^®^ XL CLIA Murex Assay, respectively (n = 1500). We also found that 03 samples were discordant for HBsAg detection between Abbott ARCHITECT^®^ system and LIAISON^®^ XL CLIA Murex Assay. DNA of HBV was extracted and PCR performed on those samples which were positively detected on the LIAISON^®^ XL CLIA Murex Assay (Table 4).

**Table 4:** Overall results of LIAISON^®^ XL CLIA Murex Assay (n = 4500).

Diasorin Method	Total	PCR	
		Positive	Negative
**Reactive**	119	119	00
**Non-Reactive**	4381	00	4381
**Total**	4500	119	4381
		**Value**	**95% CI**
**Sensitivity**		100.00%	96.95% to 100.00%
**Specificity**		100.00%	99.92% to 100.00%
**Positive Predictive Value**		100.00%	96.87% to 100%
**Negative Predictive Value**		100.00%	99.91% to 100%
**Positive Likelihood ratio**		Infinity	-,-
**Negative Likelihood ratio**		0.0	-,-
**Prevalence**		2.64%	2.20% to 3.16%
**Accuracy**		100%	

The PCR results also confirmed the presence of HBV DNA in all samples which were detected positive by LIAISON^®^ XL CLIA Murex Assay.

Even discrepancies were observed between samples detected positive by SD Bioline and GB HBsAg ELISA with 1.20%, for both methods of the samples positive for HBV infection instead of the expected 1.73% and 1.30%, respectively as confirmed by PCR (Table 5).

**Table 5:** Comparison of four assays for detection of HBV-DNA.

Assay	No of Samples Tested	Positive Detections	HBV-DNA PCR Positive	(False +ve)
**Comparison between CLIA Murex Assay and SD Bioline Rapid**				
CLIA	1500	58	58	NIL
Rapid	1500	27	17	10
**Comparison between CLIA Murex Assay and ELISA Kit**				
CLIA	1500	32	32	NIL
ELISA	1500	20	14	06
**Comparison between CLIA Murex Assay and ARCHITECT SYSTEM**				
CLIA	1500	33	33	NIL
ARCHITECT	1500	30	30	NIL

The NPV for SD Bioline, ELISA, Abbott ARCHITECT^®^ and LIAISON^®^ XL CLIA Murex Assay was 96.74%, 98.78%, 99.76% and 100%, respectively. The PPV for SD Bioline, ELISA, Abbott ARCHITECT^®^ and LIAISON^®^ XL CLIA Murex Assay was 37.04%, 70%, 100% and 100% respectively. The positive likelihood ratio for SD Bioline, ELISA, Abbott ARCHITECT^®^ and LIAISON^®^ XL CLIA Murex Assay was 14.62%, 107.4%, infinity and infinity respectively. The negative likelihood ratio for SD Bioline, ELISA, Abbott ARCHITECT^®^ and LIAISON^®^ XL CLIA Murex Assay was 0.84%, 0.56%, 0.09% and 0.00%. The prevalence was 3.87%, 2.13%, 2.20% and 2.64% respectively for SD Bioline, ELISA, Abbott ARCHITECT^®^ and LIAISON^®^ XL CLIA Murex. The accuracy for SD Bioline, ELISA, Abbott ARCHITECT^®^ and LIAISON^®^ XL CLIA Murex Assay was 95.66%, 98.40%, 99.80% and 100% respectively.

## Discussion

We performed a comparative analysis of three commonly applied HBV detection methods with the LIAISON^®^ XL Murex HBsAg Quant CLIA Assay. Hepatitis B infection is a serious health issue, worldwide, which can result in end-stage liver diseases and hepatocellular carcinoma. World Health Organization (WHO) reported that more than 240 million people have acquired HBV infections during their lives and are likely to become carriers of the virus. Among these, about 350 million remain infected chronically. It is estimated that every year more than 4 million people suffer from acute HBV [14]. Pakistan is highly endemic with HBV infection and the infection rate is increasing [15]. Approximately 3% of the population (over 6 million people) are infected, however, in some towns and villages of Sind and Punjab provinces, the infection rate is up to 20% [16, 17]. Although many measures have been taken to control the growing rate of HBV infection in Pakistan, including mandatory immunization of new born children, HBV prevalence continues to rise. Consideration of all factors involved in rising prevalence of HBV demands serious and urgent attention. The path of viral infection must be traced to reduce the rising threat. Contaminated blood transfusion appears to be one of major causes for the spread of this viral infection, and the WHO recommends that all blood donations and their products be tested for hepatitis B and other infections [18]. During 2007–08, the Pakistan Medical Research Council conducted a survey to determine prevalence of hepatitis in the Pakistan, and reported a HBV prevalence of 2.5% [19].

Previous studies have demonstrated the occurrence of HBV in Pakistan to be around 4% among the general public. In a retrospective study (2005–13) conducted in Islamabad, 2.35% of blood donors were found to be infected with HBV [2] while in 1998, Hussain *et al*., [20] found that the rate of HBV infection was 7.8% in healthy donors, with male to female ratio of 7:1. In another study, the occurrence of HBV carriers was found to be 2.9% amongst in habitants of Bahawalpur [21]. A study conducted in Rawalpindi, Northern Pakistan, indicated a HBV prevalence of 3.30% among healthy blood donors [22] while in healthy blood donors from Lahore and Faisalabad, the prevalence of HBV was found to be 2.04% and 2.06% respectively [23]. In a study comparing healthy subjects and patients with liver disease, the prevalence of hepatitis B antigen and antibody was found to be 2.90% and 35.00%, respectively, while patients with acute viral hepatitis, liver cirrhosis and hepatocellular carcinoma (HCC) indicated a prevalence of HBV 33.00%, 20.00% and 10.00%, respectively [24]. A study conducted in Karachi found 2% occurrence of HBV infection in healthy blood donors [25] and a review of 14 studies conducted in Pakistan on healthy blood donors found the frequency of HBV ranged from 3.93% SD 1.58%. A prevalence of 2.40–20.00% of HBsAg was found in health care workers and among them, the highest prevalence of HBV was seen in sweepers (20%) and dentists (17%) [26]. However, in the current study, we demonstrated an overall prevalence of 2.65% in samples positive with HBsAg. These findings thus indicate a rise in the prevalence of HBV infections.

As it has already been described earlier that rate of mutation in HBV is very high and HBsAg which is the most common target in diagnosis tests. From a diagnostic point of view, a 226 amino acids (aa) long; small hepatitis B surface antigen (sHBsAg) protein is the most important and is a major structural protein of the hepatitis B virus envelope. The structure of HBsAg is complex having discontinuous epitopes [19, 27]. From positions 100 to 160, the HBsAg amino acid sequence hydrophilic domain is designated as “a” determinant. The “a” determinants region are mainly involved in binding of antibody against HBsAg (specifically designed to bind second loop of this determinant: aa 139–147 [28,29]. HBsAg mutants were first discovered in a child born to a HBV-positive mother [30].

Many studies show that as a result of the mutation, variations occur in these conformational epitopes and, consequently, the routine diagnostic reagents are unable to detect the virus, which leads to false negative results [10,31]. In most regions of world, the presence of these mutants in carriers of HBV is unknown. In most cases, researchers are reporting false-negative results due to the presence of HBsAg mutants which cannot be detected in routine screening. Therefore, it is recommended that laboratory workers performing HBsAg detection assays must be fully educated of a given test capacity to detect mutants [32]. There was a neonatal breakthrough of HBsAg mutant infection in Singapore and investigations indicated an overall mutant prevalence of 4.6% in the country [33]. In Taiwan, a screening program was conducted for school age children and a prevalence of 0.7% in patient samples with “a” determinant mutants was found [34]. Certain HBsAg mutant carriers were also detected when a study was carried out on personal belongings of HBV chronic infection patients [35]. In Italy, the prevalence of a particular mutation in the detection site was 3.1% [36]. A study conducted in China included adults who were already vaccinated against HBV and found a 3.4% failure rate in vaccinated subjects [37]. The vaccines against HBV have provided a great protection and great many people are supposedly protected from the threat of HBV infection. However, the presence of mutants which is not detected through routine tests may increase the rate of false negatives, leading to an epidemic if not diagnosed on time [38].

Failure to detection HBV in blood donations may result in the transfusion of hepatitis B infection from donor to recipient. In the current study, we also showed that HBV detection using SD Bioline rapid and GB ELISA testing was actually only 1.20% for both tests, instead of the expected 1.73% and 1.30%, respectively as confirmed by PCR. This false positive detection data highlights another concern regarding transmission of HBV through blood transfusion. We know that whenever blood is donated, it is further divided in three parts i.e. red blood cells (RBCs), platelets and plasma. If there is a failures of HBV detection during the routine screening of donated blood samples, then it is obvious from single transfusion at least three people may become infected from the blood donation products. This increases the risk that the recipient will carry and spread the disease and may imply a great threat to health of recipient.

The SD Bioline rapid detection method showed a higher rate of discrepancies, both false negative and false positive results, when compared to the other methods under investigation. This raises some serious concerns in the use of the rapid kit detection method. The ELISA kit method showed higher specificity as compared to the SD Bioline rapid method, but lower than LIAISON^®^XL. Results from the current study reveal that the routinely used methods for HBV detection in Pakistan are not as accurate as compare to LIAISON^®^XL and may offer an escape route to mutant and wild type HBV. Mutant HBV may remain undetected if these methods are continued, resulting in the easy and rapid transfer of the virus.

There is a great need to implement a unanimous policy regarding blood donation and transfusion in Pakistan. Currently, different types of HBV testing kits are being used both in government and private sectors. The quality kits must be used across the country under a policy to detect the wild and mutants HBsAg, which are not accurately detected by currently used methods.

There is no central quality control and management regarding the types, sensitivity & credibility of detection methods. In the market, there is no information on how many low quality devices/kits are being used for HBV detection and screening. For this study, it is clear that both wild and mutant viruses may be escaping due to lack of quality control and policy. This sector requires urgent attention from the concerned authorities.

Krawczyk A. *et*. *al*., evaluated the capability of LIAISON^®^ XL Murex HBsAg Quant Assay to detect various HBsAg mutants that were previously described in terms of immune and detection escape (aa118–147) or in terms of drug resistance (aa161–196). All mutants were detected. Moreover, they mentioned an excellent specificity and agreement between the Abbott ARCHITECT^®^ and the fully automated and closed analyzer LIAISON^®^XL for the detection of HBsAg [39]. Similar results were obtained by Malm K. *et*. *al*., on a blood donor population [40]. A study conducted by Ghisetti V. *et*. *al*., also found that the DiaSorin LIAISON^®^ HBsAg assay shows excellent standardization according to standards established by the WHO. This assay is appropriate for clinical laboratory and blood bank practice [41]. In line with the existing literature, the present study, also determined that LIAISON^®^XL has best detection capabilities than any of the commonly used routine detection methods.

The HBV screening kits available and utilized for screening donors in the blood banks in Pakistan have a wide range of variability in their specificity and sensitivity. Mutant HBV may escape routine diagnostic assays and may be transmitted to healthy individuals causing acute and chronic infection. The routine transfusion detection tests, usually based on the SD Bioline rapid or ELISA, must be revised. In contrast, the LIAISON^®^XL murex HBsAg assay is a highly specific, sensitive and accurate screening assay for detecting wild type as well as mutant HBV. There is a need to use only high quality detection kits and reagents, for screening to curtail the spread of HBV. A policy should be formulated at the national level with country wide implementation regarding the selection of standard detection method and kits for HBV screening purposes. The policy should clearly outline the criteria of standard of kits regarding detection of wild and mutant type of viruses. Molecular studies are needed to determine the exact mutation in surface antigen. New guidelines for diagnosis and management of HBsAg mutant infection are also required. This may include a study national level to find the type of prevailing mutants that may help to explicate the perspective of HBsAg mutants to make diagnostics and blood transfusion safe. Considering its detection performance, the use of LIAISON^®^XL Murex HBsAg Quant assay may help to reach this goal reliably and rapidly.

## Supporting information

S1 Table Comparison of four assays for detection of HBV DNA. (DOCX)

S2 Table Overall results of Abbott. (DOCX)

S3 Table Overall results of LIAISON^®^ XL CLIA Murex Assay. Renamed_f48cd. (DOCX)

S4 Table Overall results of rapid test. (DOCX)

S5 Table Overall results of ELISA. (DOCX)

S6 Table Results file for statistics. (DOCX)
