# Supplementary material for: Detection of HBsAg mutants in the blood donor population of Pakistan
Source: PLoS One. 2017 Nov 22;12(11):e0188066. doi: 10.1371/journal.pone.0188066 (PMC5699832; doi:10.1371/journal.pone.0188066)
Supplement: S1 Table — (DOCX) [file pone.0188066.s001.docx]

| **Table 5: Comparison of four assays for detection of HBV DNA** | | | | |
| --- | --- | --- | --- | --- |
| **Assay** | **No of Samples tested** | **Positive Detections** | **HBV-DNA PCR positive** | **(false +ve)** |
| Comparison between CLIA Murex Assay and SD Bioline Rapid | | | | |
| CLIA | 1500 | 58 | 58 | NIL |
| Rapid | 1500 | 27 | 17 | 10 |
| Comparison between CLIA Murex Assay and ELISA Kit | | | | |
| CLIA | 1500 | 32 | 32 | NIL |
| ELISA | 1500 | 20 | 14 | 06 |
| Comparison between CLIA Murex Assay and ARCHITECT SYSTEM | | | | |
| CLIA | 1500 | 33 | 33 | NIL |
| ARCHITECT | 1500 | 30 | 30 | NIL |
