# Supplementary material for: Detection of HBsAg mutants in the blood donor population of Pakistan
Source: PLoS One. 2017 Nov 22;12(11):e0188066. doi: 10.1371/journal.pone.0188066 (PMC5699832; doi:10.1371/journal.pone.0188066)
Supplement: S2 Table — (DOCX) [file pone.0188066.s002.docx]

| **Table - 3: Overall results of Abbott Architect^®^ (n=1500)** |
| --- |

| **Abbot METHOD** | **TOTAL** | **PCR** |
| --- | --- | --- |

|  |  | **Positive** | **Negative** |
| --- | --- | --- | --- |

| **Reactive** | 30 | 30 | 00 |
| --- | --- | --- | --- |
| **Non-Reactive** | 1470 | 03 | 1467 |
| **TOTAL** | 1500 | 33 | 1467 |
|  | | | |

|  | **Value** | **95% CI** |
| --- | --- | --- |
| **Sensitivity** | 90.91% | 75.67% to 98.08% |
| **Specificity** | 100.00 % | 99.75% to 100.00% |
| **Positive Predictive Value** | 100.00% | 88.64% to 100% |
| **Negative Predictive Value** | 99.80 % | 99.40% to 99.93% |
| **Positive Likelihood ratio** | infinity |  |
| Negative Likelihood ratio | 0.09 | 0.03 to 0.27 |
| Prevalence | 2.20% | 1.52% to 3.08% |
| Accuracy | 99.8% | |
