# Supplementary material for: Detection of HBsAg mutants in the blood donor population of Pakistan
Source: PLoS One. 2017 Nov 22;12(11):e0188066. doi: 10.1371/journal.pone.0188066 (PMC5699832; doi:10.1371/journal.pone.0188066)
Supplement: S3 Table — (DOCX) [file pone.0188066.s003.docx]

| **Table - 4: Overall results of LIAISON^®^ XL CLIA Murex Assay (n=4500)** | | |
| --- | --- | --- |
| **Diasorin Method** | **Total** | **PCR** |

|  |  | **Positive** | **Negative** |
| --- | --- | --- | --- |

| **Reactive** | 119 | 119 | 00 |
| --- | --- | --- | --- |
| **Non-Reactive** | 4381 | 00 | 4381 |
| **Total** | 4500 | 119 | 4381 |
|  | | | |

|  | **Value** | **95% CI** |
| --- | --- | --- |
| **Sensitivity** | 100.00% | 96.95% to 100.00% |
| **Specificity** | 100.00 % | 99.92% to 100.00% |
| **Positive Predictive Value** | 100.00% | 96.87% to 100% |
| **Negative Predictive Value** | 100.00 % | 99.91% to 100% |
| **Positive Likelihood ratio** | Infinity |  |
| Negative Likelihood ratio | 0.0 |  |
| Prevalence | 2.64% | 2.20% to 3.16% |
| Accuracy | 100% | |
