# Supplementary material for: Detection of HBsAg mutants in the blood donor population of Pakistan
Source: PLoS One. 2017 Nov 22;12(11):e0188066. doi: 10.1371/journal.pone.0188066 (PMC5699832; doi:10.1371/journal.pone.0188066)
Supplement: S4 Table — (DOCX) [file pone.0188066.s004.docx]

| **Table - 1: Overall results of SD Bioline Rapid KIT(n=1500)** | | |
| --- | --- | --- |
| **KIT METHOD**  **SD Bioline Rapid** | **TOTAL** | **PCR** |

|  |  | | **Negative** | | **Negative** |
| --- | --- | --- | --- | --- | --- |
| **Reactive** | 27 | | 10 | | 17 |
| **Non-Reactive** | 1473 | | 48 | | 1425 |
| **TOTAL** | 1500 | | 58 | | 1432 |
|  | | | | | |
|  | | **Value** | | **95% CI** | |
| **Sensitivity** | | 17.24% | | 8.59% to 29.43% | |
| **Specificity** | | 98.82 % | | 98.12% to 99.31% | |
| **Positive Predictive Value** | | 37.04% | | 21.99% to 55.11% | |
| **Negative Predictive Value** | | 96.74 % | | 96.35% to 97.09% | |
| **Positive Likelihood ratio** | | 14.62 | | 7.01 to 30.52 | |
| Negative Likelihood ratio | | 0.84 | | 0.74 to 0.94 | |
| Prevalence | | 3.87% | | 2.95% to 4.97% | |
| Accuracy | | **95.66%** | | | |
