# Supplementary material for: Detection of HBsAg mutants in the blood donor population of Pakistan
Source: PLoS One. 2017 Nov 22;12(11):e0188066. doi: 10.1371/journal.pone.0188066 (PMC5699832; doi:10.1371/journal.pone.0188066)
Supplement: S5 Table — (DOCX) [file pone.0188066.s005.docx]

| **Table - 2: Overall results of ELISA (n=1500)** |
| --- |

| **ELISA METHOD** | **TOTAL** | | **PCR** | | |
| --- | --- | --- | --- | --- | --- |
|  |  |  | **Positive** | | **Negative** |
| **Reactive** | 20 | | 14 | | 06 |
| **Non-Reactive** | 1480 | | 18 | | 1462 |
| **TOTAL** | 1500 | | 32 | | 1468 |
|  | | | | | |
|  | | **Value** | | **95% CI** | |
| **Sensitivity** | | 43.75% | | 26.36% to 62.34% | |
| **Specificity** | | 99.59 % | | 99.11% to 99.85% | |
| **Positive Predictive Value** | | 70.00% | | 48.93% to 85.03% | |
| **Negative Predictive Value** | | 98.78 % | | 98.36% to 99.10% | |
| **Positive Likelihood ratio** | | 107.04 | | 43.96 to 260.65 | |
| Negative Likelihood ratio | | 0.56 | | 0.42 to 0.77 | |
| Prevalence | | 2.13% | | 1.46% to 3.00% | |
| Accuracy | | 98.4% | | | |
