# Supplementary material for: Detection of HBsAg mutants in the blood donor population of Pakistan
Source: PLoS One. 2017 Nov 22;12(11):e0188066. doi: 10.1371/journal.pone.0188066 (PMC5699832; doi:10.1371/journal.pone.0188066)
Supplement: S6 Table — (DOCX) [file pone.0188066.s006.docx]

**Total % age of HBV 2.64**

**Rapid**

Total samples: 1500

Positive: 27+ False Positive **17**

True Positive (PCR): 58

Discordant samples: 31

| **KIT METHOD**  **SD Bioline Rapid** | **TOTAL** | **PCR** | | **PPV** | **NPV** | **Sensitivity** | **Specificity** |
| --- | --- | --- | --- | --- | --- | --- | --- |
|  |  | **Positive** | **Negative** |  |  |  |  |
| **Reactive** | 27 | 10 | 17 | 37.04% | 96.74% | 17.24% | 98.82% |
| **Non-Reactive** | 1473 | 48 | 1425 |  |  |  |  |
| **TOTAL** | 1500 | 58 | 1432 |  |  |  |  |

| **Statistic** | **Value** | **95% CI** |
| --- | --- | --- |
| **Sensitivity** | 17.24% | 8.59% to 29.43% |
| **Specificity** | 98.82 % | 98.12% to 99.31% |
| **Positive Predictive Value** | 37.04% | 21.99% to 55.11% |
| **Negative Predictive Value** | 96.74 % | 96.35% to 97.09% |

**ELISA**

Total samples: 1500

Positive: 20 + False Positive **06**

True Positive (PCR): 32

Discordant samples: 12

| **ELISA METHOD** | **TOTAL** | **PCR** | | **PPV** | **NPV** | **Sensitivity** | **Specificity** |
| --- | --- | --- | --- | --- | --- | --- | --- |
|  |  | **Positive** | **Negative** |  |  |  |  |
| **Reactive** | 20 | 14 | 06 | 70% | 98.78% | 43.75% | 99.59% |
| **Non-Reactive** | 1480 | 18 | 1462 |  |  |  |  |
| **TOTAL** | 1500 | 32 | 1468 | - | - | - | - |

| **Statistic** | **Value** | **95% CI** |
| --- | --- | --- |
| **Sensitivity** | 43.75% | 26.36% to 62.34% |
| **Specificity** | 99.59 % | 99.11% to 99.85% |
| **Positive Predictive Value** | 70.00% | 48.93% to 85.03% |
| **Negative Predictive Value** | 98.78 % | 98.36% to 99.10% |

**Abbott:**

Total samples: 1500

Positive: 30

True Positive (PCR): 33

Discordant samples: 03

| **Abbot METHOD** | **TOTAL** | **PCR** | | **PPV** | **NPV** | **Sensitivity** | **Specificity** |
| --- | --- | --- | --- | --- | --- | --- | --- |
|  |  | **Positive** | **Negative** |  |  |  |  |
| **Reactive** | 30 | 30 | 00 | 100% | 99.79% | 90.9% | 100% |
| **Non-Reactive** | 1470 | 03 | 1467 |  |  |  |  |
| **TOTAL** | 1500 | 33 | 1467 | - | - | - | - |

| **Statistic** | **Value** | **95% CI** |
| --- | --- | --- |
| **Sensitivity** | 90.91% | 75.67% to 98.08% |
| **Specificity** | 100.00 % | 99.75% to 100.00% |
| **Positive Predictive Value** | 100.00% |  |
| **Negative Predictive Value** | 99.80 % | 99.40% to 99.93% |

**DiaSorin XL:**

Total samples: 4500

Positive: 119

True Positive (PCR): 119

Discordant samples: nil

| **Diasorin METHOD** | **TOTAL** | **PCR** | | **PPV** | **NPV** | **Sensitivity** | **Specificity** |
| --- | --- | --- | --- | --- | --- | --- | --- |
|  |  | **Positive** | **Negative** |  |  |  |  |
| **Reactive** | 119 | 119 | 00 | 100% | 100% | 100% | 100% |
| **Non-Reactive** | 4381 | 00 | 4381 |  |  |  |  |
| **TOTAL** | 4500 | 119 | 4381 |  |  | - | - |

| **Statistic** | **Value** | **95% CI** |
| --- | --- | --- |
| **Sensitivity** | 100.00% | 96.95% to 100.00% |
| **Specificity** | 100.00 % | 99.92% to 100.00% |
| **Positive Predictive Value** | 100.00% |  |
| **Negative Predictive Value** | 100.00 % |  |
